# Beyond performance: the role of task demand, effort, and individual differences in ab initio pilots

**DOI:** 10.1038/s41598-023-41427-4

**Published:** 2023-08-28

**Authors:** Mohammad-Javad Darvishi-Bayazi, Andrew Law, Sergio Mejia Romero, Sion Jennings, Irina Rish, Jocelyn Faubert

**Affiliations:** 1 Faubert Lab, Université de Montréal, Montréal, QC Canada; 2 Mila-Québec AI Institute, Montréal, QC Canada; 3 Université de Montréal, Montréal, QC Canada; 4 National Research Council Canada, Ottawa, ON Canada

**Keywords:** Neuroscience, Cognitive neuroscience, Emotion, Stress and resilience, Aerospace engineering, Biomedical engineering

## Abstract

Aviation safety depends on the skill and expertise of pilots to meet the task demands of flying an aircraft in an effective and efficient manner. During flight training, students may respond differently to imposed task demands based on individual differences in capacity, physiological arousal, and effort. To ensure that pilots achieve a common desired level of expertise, training programs should account for individual differences to optimize pilot performance. This study investigates the relationship between task performance and physiological correlates of effort in ab initio pilots. Twenty-four participants conducted a flight simulator task with three difficulty levels and were asked to rate their perceived demand and effort using the NASA TLX. We recorded heart rate, EEG brain activity, and pupil size to assess changes in the participants’ mental and physiological states across different task demands. We found that, despite group-level correlations between performance error and physiological responses, individual differences in physiological responses to task demands reflected different levels of participant effort and task efficiency. These findings suggest that physiological monitoring of student pilots might provide beneficial insights to flight instructors to optimize pilot training at the individual level.

## Introduction

Flight safety heavily depends on proper pilot training to prevent or minimize human error^1,2^, which is the cause of nearly 70$\%$ of aviation accidents^3^. During training, students undergo a series of learning and practice exercises to develop the knowledge and skills required to perform progressively more difficult tasks with desired performance outcomes. Throughout this process, the student may encounter task demands that either require significant effort to perform or are beyond their current capacity. This may cause an increase in physiological arousal in response to perceived demands, which is also influenced by the history of success or failure on previous attempts.

The relationship between task demands and performance is complex and multi-faceted^4–6^. When arousal is at an optimal level and task demands are reasonable, peak performance can be achieved with the appropriate level of effort. But if arousal gets too high or perceived task demands exceed capacity, the student may disengage from the task and withdraw effort, resulting in poor performance. As a student develops skill-based expertise, the level of mental effort required to perform a specific task often decreases, opening up the capacity to take on additional demands. To know when a student has achieved a desired level of expertise, it would be helpful to track changes in effort across task demands and performance outcomes. Whereas imposed task demands and performance outcomes can be defined or quantified concretely, effort can only be inferred indirectly using subjective ratings, behavioural metrics, or psychophysiological measures.

Subjective rating questionnaires, such as the NASA Task Load Index (TLX)^7^ or Bedford workload scale^8^, allow users to self-report their perceived task demands, effort, workload, and/or spare capacity. However, these qualitative data are generally collected post hoc, are unable to capture time-resolved changes in effort during task performance, and may therefore reflect the most salient moments of workload or effort during the task. Metrics derived from flight control activity can provide insights into the amplitude and frequency of control movements, but may not be sensitive to changes in mental effort underlying the presence or absence of control movement.

By comparison, non-invasive measures of brain and cardiac function provide accessible means of detecting changes in cognitive and physiological state in a time-resolvable manner. Examples include pupillometry, electrodermal activity (EDA), electrocardiography (ECG)^9^, electroencephalography (EEG)^10,11^, and functional near-infrared spectroscopy (fNIRS)^12^, which have been applied in laboratory^13^, virtual reality^11^, flight simulator^14^, and real flight environments^15^. Neurophysiological measures are also used for brain-computer interfaces (BCI) to restore or enhance human performance across many domains. One specific area of BCI research is the prediction of cognitive states (e.g., workload) from neurophysiological data, often using imposed task demands as ground truth labels. In complex operational environments, however, these prediction algorithms often fail to scale and generalize well due to significant individual differences in physiological responses to task demands.

While problematic for BCI prediction algorithms, individual differences in physiological response and task performance may provide important insights into how students are progressing through a training program. Neural efficiency is defined as the ratio between task performance and effort^16^. In the training context, a student is likely to progress from a state of lower efficiency (high effort but poor performance) to higher efficiency (low effort and good performance). While effort is not directly quantifiable, multiple physiological measures have been shown to correlate with effort, including heart rate^17^, EEG frontal theta^18^, and pupil size^19^.

Here, we investigate how subjective ratings of demand and effort change with task difficulty level during ab initio pilot training, and how these changes are reflected in performance and physiological responses. We show that, whereas group-level differences in the physiological state may correlate with changes in performance due to task difficulty, individual physiological responses are highly variable and may provide additional insight into the level of effort underlying performance that could be used to improve training.

## Results

In this study, we recruited twenty-four participants to perform basic instrumentation flying maneuvers in a flight simulator with three difficulty levels (D1, D2, and D3). D1 maneuvers involved a single-direction change in one axis (altitude or heading), D2 maneuvers involved a change with reversal in one axis (altitude or heading), and D3 maneuvers involved simultaneous changes in both axes (altitude and heading) pairing a single-direction change in one axis with a reversal in the other. Participants completed video-based tutorials and hands-on training exercises prior to undertaking a set of 15 maneuver trials which were counter-balanced across difficulty levels. The participants rated their workload following each trial using the NASA Task Load Index (TLX) questionnaire^7^. Throughout each trial, yoke position, aircraft state (heading, altitude, etc.), and physiological responses (ECG, EEG, and pupillometry) were recorded. A detailed description of the experimental protocol and data processing methodology is provided in the “Methods” section.

### NASA TLX workload ratings

**Figure 1:**
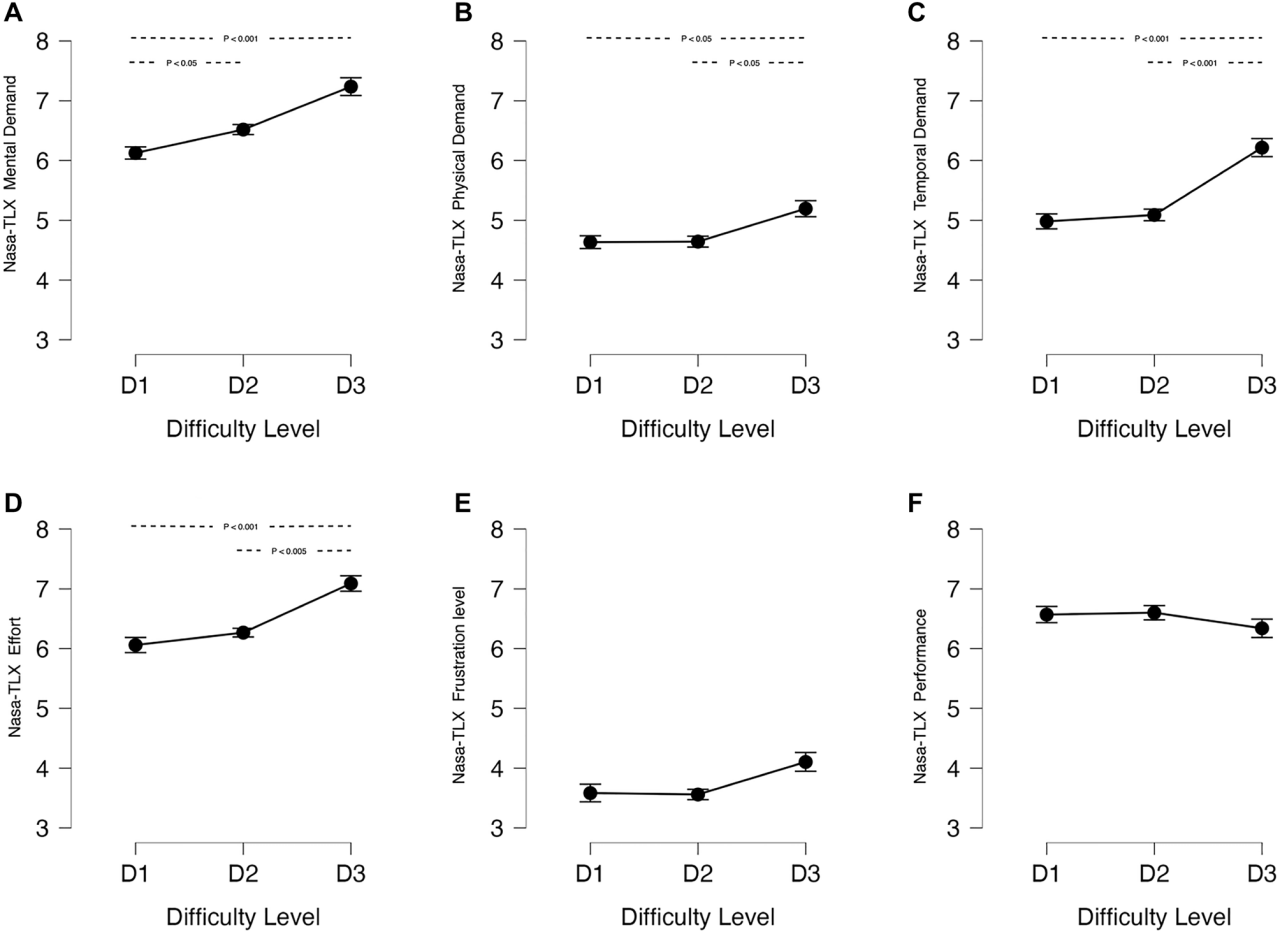
Effect of task difficulty level on NASA-TLX sub-scale ratings. Error bars represent the standard error.

In general, the participants rated mental demand and effort the highest, followed by temporal demand, physical demand, and frustration (Fig. 1). Maneuver difficulty had a significant effect on mental demand ($X^2$(2) = 17.426, p < 0.001), physical demand ($X^2$(2) = 9.53, p = 0.009), temporal demand ($X^2$(2) = 20.33, p < 0.001), and effort ($X^2$(2) = 21.00, p < 0.001). For all four of these sub-scales, ratings for D3 were significantly higher than D1 ($p_{holm}<0.005$). Ratings of mental demand were higher for D2 than D1 ($p_{holm}<0.05$), but not significantly different between D2 and D3, whereas ratings of temporal demand, physical demand, and effort were higher for D3 than D2, but not different between D2 and D1. Interestingly, ratings of frustration and performance were similar across difficulty levels. A closer look at performance ratings revealed that nearly half of the participants incorrectly interpreted the scale and rated better performance closer to 10 and worse performance closer to 1. As such, group-level performance ratings were not a reliable indicator of overall performance differences between maneuver difficulty levels and were not included in further analysis.

### Maneuver performance

**Figure 2:**
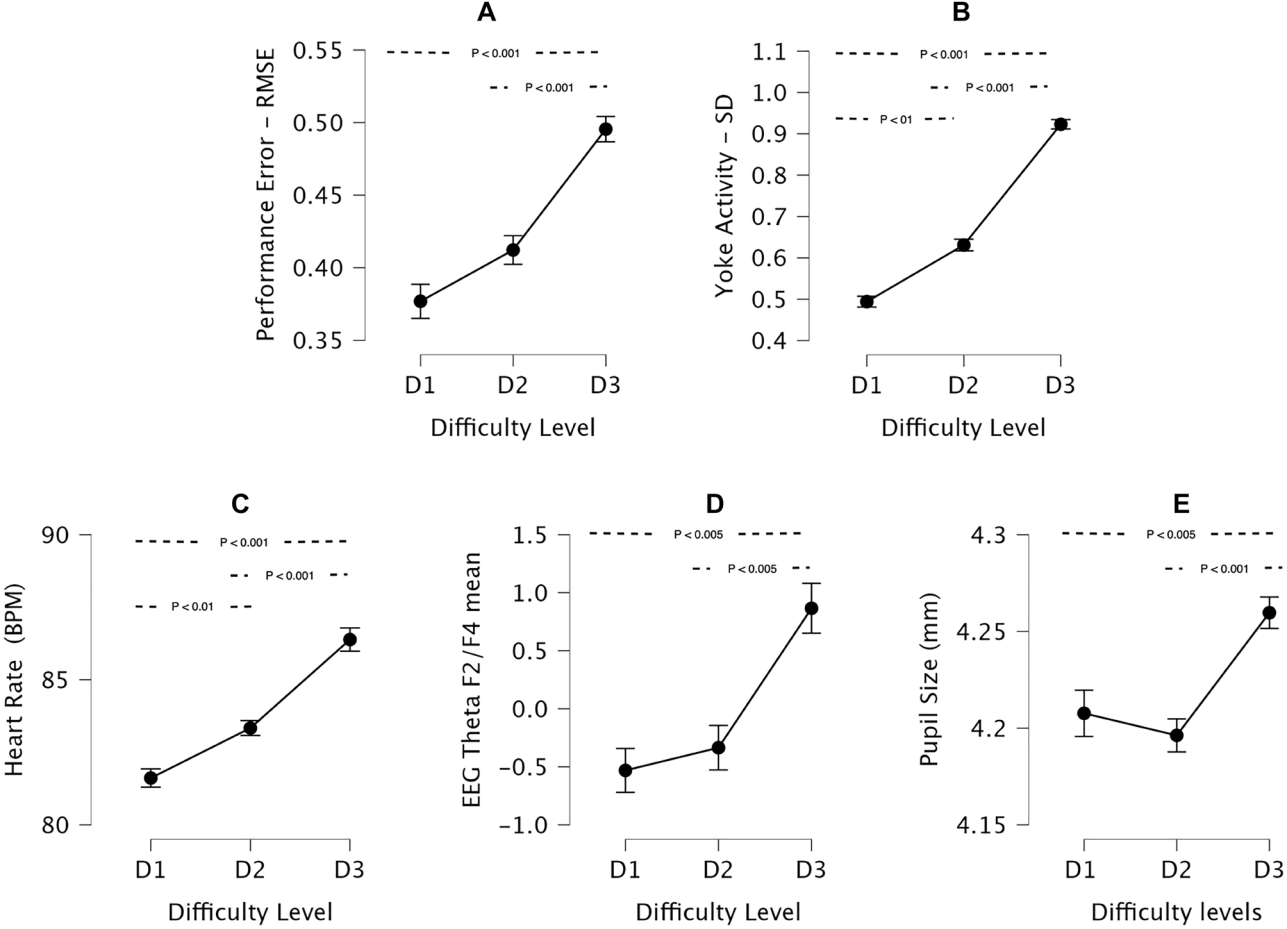
Effects of task difficulty level on control activity, performance, and physiological responses. Error bars represent the standard error.

Maneuver performance was quantified as the root mean square error (RMSE) between the actual heading and altitude for each trial and the idealized heading and altitude time-history for that specific maneuver instruction (Fig. 2A). RMSE showed a significant increase with difficulty level ($X^2$(2) = 29.25, p < 0.001), with higher errors during D3 maneuvers compared to D2 and D1 ($p_{holm}<0.001$), but no significant difference between D2 and D1 maneuvers.

### Control activity

Participant control activity was quantified for each trial as the standard deviation (SD) of the yoke position (Fig. 2B). Yoke positions for roll and pitch axes were normalized separately, relative to full-scale deflection, and overall control activity was defined as the root mean square SD across both axes. Manuever difficulty had a significant effect on yoke SD ($X^2$(2) = 44.33, p < 0.001), with higher control activity observed for D3 compared to D2 and D1 ($p_{holm}<0.001$), and for D2 compared to D1 ($p_{holm}=0.006 < 0.01$).

### Heart rate

ECG recordings were processed to derive the participants’ mean heart rate (HR) for each maneuver trial. HR increased significantly with maneuver difficulty (Fig. 2C; ($X^2$(2) = 44.33, p < 0.001); higher HR was observed for D3 compared to D2 and D1 ($p_{holm}<0.001$) and for D2 compared to D1 ($p_{holm}=0.006 < 0.01$).

### EEG frontal theta

EEG theta band power was computed from 64-channel EEG recordings. For simplicity, we evaluated the average theta band-power across frontal channels F2 and F4 (i.e. EEG frontal theta), which have previously been shown to correlate with task difficulty level^20–22^. EEG frontal theta showed a significant increase with maneuver difficulty (Fig. 2D; ($X^2$(2) = 14.08, p < 0.001), with higher levels of theta observed during D3 than D2 and D1 ($p_{holm}<0.005$), but no significant difference between D2 and D1.

### Pupil size

Pupil size was measured using a Fovio FX3 eye tracker. For a subset of participants and trials, pupil size data was invalid or unavailable due to interruptions in continuous eye tracking (see “Methods” section). For the pupil size data that was available, there was a significant effect of maneuver difficulty (Fig. 2E; ($X^2$(2) = 18.58, p < 0.001), with larger pupil sizes for D3 than D2 ($p_{holm}<0.001$), and significant difference between D1 versus D3 (($p_{holm}<0.005$) but no significant difference between D2 and D1.

### Correlation of measures

Pairwise repeated measures correlations were computed between all of the above measures except for NASA TLX Frustration and Performance ratings, which did not show a significant effect of maneuver difficulty. In all cases, pairwise correlations were found to be statistically significant, though the coefficient of determination varied widely (see Supplementary Fig. S1–S3). Interestingly, the NASA TLX sub-scale ratings were all more highly correlated with control activity than any other objective measure (e.g., mental demand $R^{2} = 0.54$). Performance RMSE ($R^{2} = 0.58$), heart rate ($R^{2} = 0.69$), EEG theta ($R^{2} = 0.31$), and pupil size ($R^{2} = 0.30$) were also more highly correlated with control activity than any other measure. Also notable was that performance RMSE was weakly correlated with physiological measures (e.g., heart rate $R^{2} = 0.36$, EEG theta $R^{2} = 0.17$) despite similar overall trends at the group level (Fig. 2). This indicates that there were significant individual differences in performance and physiological response.

### Individual differences in performance and physiological response

To examine relative differences in performance versus physiological response at the individual level, performance RMSE was compared to EEG frontal theta and heart rate for each participant separately. (Pupil size was not included here due to incomplete data for all participants; see “Methods” section). Given that the participants reported significantly higher demand and effort for D3 maneuvers than D1 or D2, we specifically focused on changes in performance RMSE, EEG theta, heart rate from D2 to D3. Performance and physiological responses for D2 and D3 maneuvers were referenced to D1 (i.e., D2–D1 and D3–D1), to account for individual differences in D1 measures, and then z-scored normalized across participants.

**Figure 3:**
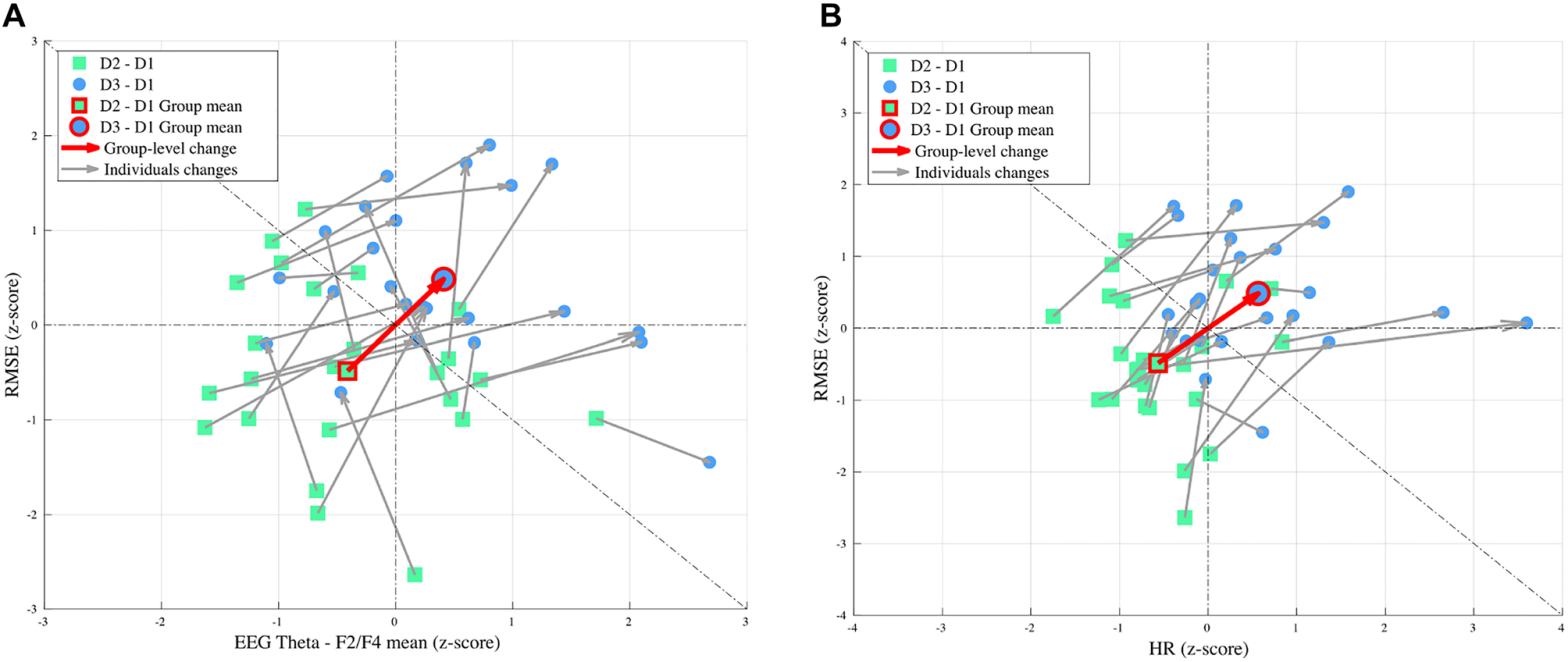
Individual differences in performance and physiological response from D2 to D3. (**A**) Z-score normalized RMSE versus EEG frontal theta. (**B**) Z-score normalized RMSE versus heart rate. Green squares indicate normalized D2 values and blue circles indicate normalized D3 values. Arrows connect normalized D2–D3 values at the individual and group-level (highlighted in red).

Normalized D2 and D3 measures were evaluated in two ways (Fig. 3): (1) RMSE versus EEG frontal theta and (2) RMSE versus heart rate. At the group level, an increase in RMSE from D2 to D3 coincided with an increase in EEG frontal theta and an increase in heart rate. This might reflect a decrease in task efficiency from D2 to D3 (i.e., a decrease in performance despite an increase in effort). However, at the individual level, there was considerable variability in performance and physiological responses going from D2 to D3. In particular, RMSE decreased from D2 to D3 for 2 participants, and EEG frontal theta decreased from D2 to D3 for 6 participants. By comparison, heart rate increased from D2 to D3 for all 24 participants. Moreover, some participants showed a larger relative change in RMSE compared to EEG frontal theta or heart rate, and vice versa. As such, group-average performance and physiological measures were generally not representative of individual responses.

### Sub-grouping by physiological response

**Figure 4:**
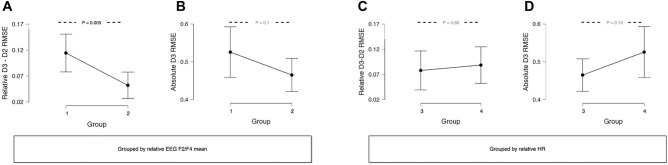
Sub-group performance based on physiological response. (**A**) Relative RMSE between groups based on EEG frontal theta (**B**) Absolute RMSE between groups based on EEG frontal theta. (**C**) Relative RMSE of two groups based on heart rate. (**D**) Absolute RMSE between groups based on heart rate.

To further examine the relation between performance and physiological response, the 24 participants were divided into two sub-groups of 12 to test for differences in task efficiency. The first sub-grouping was based on D2-to-D3 changes in EEG frontal theta (group 1: 12 participants with the lowest change in EEG frontal theta; group 2: 12 participants with the highest change in EEG frontal theta). The second sub-grouping was based on D2-to-D3 changes in heart rate (group 3: 12 participants with the lowest change in heart rate; group 4: 12 participants with the highest change in heart rate). In both cases, the sub-groups were compared for differences in relative and absolute RMSE for D3 maneuvers (Fig. 4).

Relative RMSE was significantly lower for group 2 than group 1 (p = 0.005), indicating that the participants who had a higher increase in EEG frontal theta from D2 to D3 had a smaller decrease in performance from D2 to D3. By comparison, relative RMSE was similar for groups 3 and 4, indicating that relative changes in heart rate were not consistently associated with relative changes in performance.

Absolute RMSE was not significantly different between either set of sub-groups, but interestingly, the trends were in opposite directions for EEG frontal theta and heart rate. The mean absolute RMSE was lower for group 2 than group 1, but higher for group 4 than group 3. This suggests that lower D3 RMSE (i.e., better performance) generally coincided with higher EEG frontal theta (group 2) and/or lower heart rate (group 3), whereas higher D3 RMSE (i.e., worse performance) coincided with lower EEG frontal theta (group 1) and/or higher heart rate (4). Five participants were in both groups 2 and 3, whereas 6 participants were in both groups 1 and 4.

## Discussion

This study aimed to compare the relative sensitivity of subjective ratings, objective measures, and physiological responses to changes in pilot workload during ab initio training. Rather than use physiological measures to infer or predict workload, the physiological measures were instead used to examine individual differences in task efficiency.

The group-level results (Figs. 1 and 2) demonstrate that dual-axis (D3) maneuvers were significantly more difficult than single-axis (D1 or D2) maneuvers. D3 maneuvers resulted in the highest subjective ratings of mental demand, physical demand, temporal demand, and effort, the highest level of control activity and performance error (RMSE), and the highest levels of heart rate, EEG frontal theta, and pupil size. Overall, there was a non-linear response to maneuver difficulty, with D2 responses generally similar to D1 and a substantial increase in workload going to D3. However, there were notable differences in the sensitivity of the various measures to D1 versus D2 maneuvers. Mental demand ratings showed a significant increase from D1 to D2, as did measures of control activity and heart rate. The other sub-scales and objective measures showed no significant differences between D1 and D2. This suggests that while there was a detectable change in participants’ mental and physiological state from D1 to D2, a more substantial change occurred from D2 to D3 in response to the demands of dual-axis maneuvers.

The examination of individual differences in physiological response and performance between D2 and D3 (Fig. 3) indicated that the task efficiency varied widely across participants. For some participants, a large increase in EEG frontal theta that coincided with a small change in RMSE, potentially reflecting a substantial increase in effort to complete D3 with a similar performance level to D2. While an increase in effort with constant or worse performance reflects a decrease in efficiency, this is preferable to the case where effort is withdrawn in D3 due to excessive demand. This is where physiological measures such as EEG frontal theta may benefit a pilot training program.

In the absence of physiological measures that correlate with effort, it is difficult to discern if a student’s performance decrement under higher task demand is simply due to a lack of skill (i.e., excessive effort required) or if the student has partially or completely disengaged from the task (i.e., effort withdrawn due to excessive demand). In the first scenario, the student might benefit from repeated attempts at the current level of task demand until skill-based expertise is developed and the task can be completed with lower effort. In the second scenario, the student might benefit from a temporary reduction in task demand until sufficient skills are developed to meet the current task demand without effort withdrawal.

Future studies are needed to investigate if physiological correlates of effort are sensitive to improvements in efficiency as skill develops over multiple pilot training sessions. Moreover, additional research is required to test the effects of efficiency-based modification to task demand on overall training outcomes. However, based on the results of this current study, we conclude that a flight instructor could benefit from indicators of student effort based on physiological measures to customize the training sessions and optimize pilot training and performance.

## Methods

### Sample

Twenty-four volunteers (13 women and 11 men), aged 21–41 years (Mean = 28.87, SD = 4.60), participated in this study. Most of them were right-handed (2 left-handed) and they were in good general health with normal or corrected-to-normal vision. The study was approved by the Université de Montréal ethics committee for health research ethics committees (Comité d’éthique de la recherche en santé CERES-18-135-D). The experiment followed all recommended ethical procedures and guidelines. We obtained informed consent from all participants, who were compensated for their time and travel costs.

### Flight simulator and task

**Figure 5:**
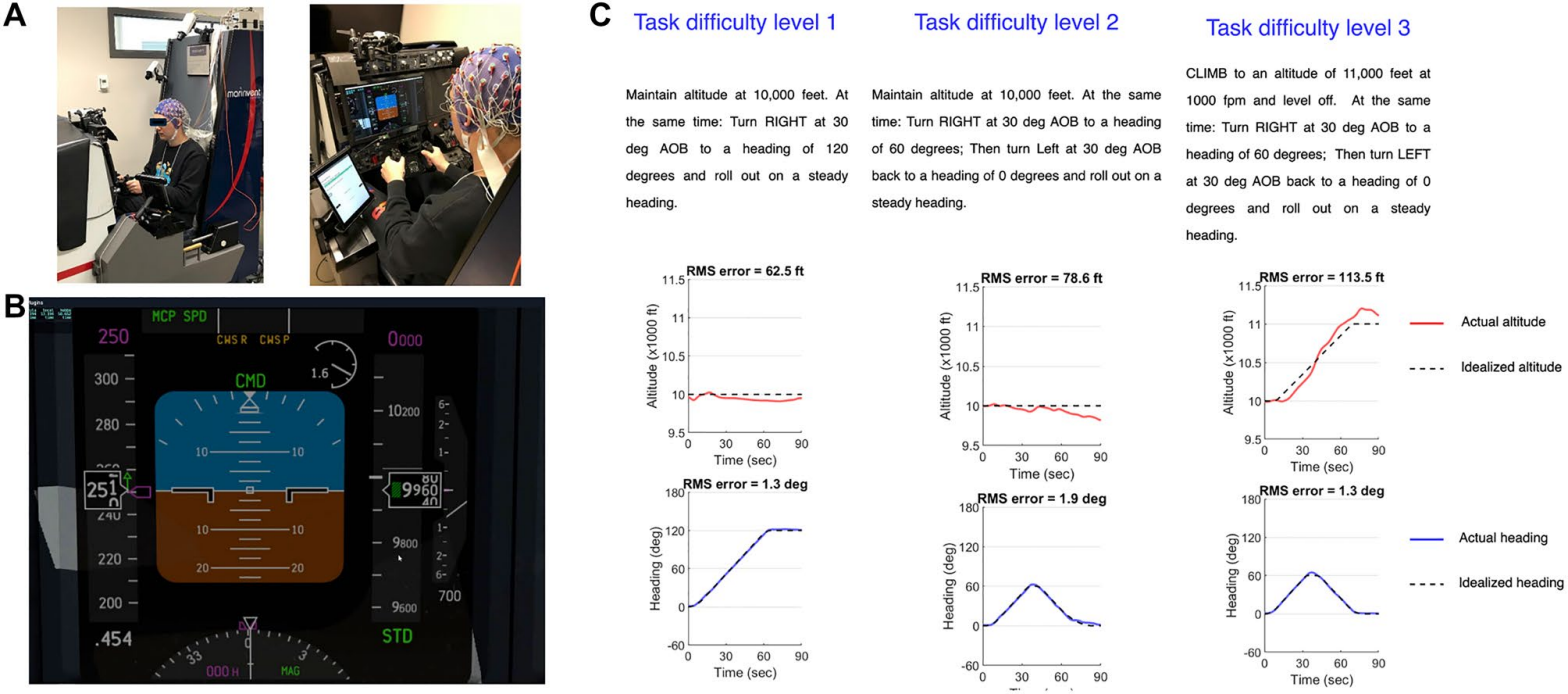
Flight simulator and Task. (**A**) A participant sitting in the simulator, (**B**) primary flight display used to complete the task. (**C**) Examples of task instructions. One example for each task difficulty level. Each task has several variants (e.g. difficulty level 1 includes turning left/right or climbing/descending). The black dashed lines show the idealized target profiles that minimize RMS error relative to the actual altitude (red) and heading (blue) for this specific trial.

We used the Marinvent flight simulator (Marinvent Corporation, QC, Canada) and X-Plane 11 software (Fig. 5B). The setup comprised one display panel (17-inch diagonal, Resolution: 1080p, updated at 60 Hz) that offered approximately a $32^{\circ }$ field of view and a tablet to present the instructions. As shown in Fig. 5A, participants sat comfortably in the flight simulator seat to see the Primary Flight Display (PFD), which provided visual cues of aircraft heading, altitude, angle of bank, and vertical speed. The tablet showed the pre-designed maneuvers by pilots with different difficulty levels. Figure 5C presents one example of each task difficulty level. Participants used the simulator yoke to control aircraft altitude and heading, while airspeed was under automated control.

Instructed maneuvers were divided into three difficult levels: D1, D2, and D3. D1 maneuvers involved a single-direction change in one axis (altitude or heading), D2 maneuvers involved a change with reversal in one axis (altitude or heading), and D3 maneuvers involved simultaneous changes in both axes (altitude and heading) pairing a single-direction change in one axis with a reversal in the other. As such, there were four variants of D1, four variants of D2, and eight variants of D3 (see Supplemental Material S1).

Each maneuver duration was 90 s. The participants also completed a baseline task of 30 s before each maneuver to stabilize and isolate different task difficulties. The baseline instruction asked participants to maintain the altitude in the initial condition. The maneuvers were divided into three blocks. The first block was considered the initial training phase and consisted of 7 trials: four D1 maneuvers followed by two D2 maneuvers followed by one D3 maneuver. The second and third blocks consisted of 15 trials, which were pseudo-randomly selected from the list of instructions such that an equal number of D1, D2, and D3 conditions were chosen. These 15 trials (five D1s, five D2s, and five D3s) were used to evaluate performance relative to physiological response.

### Recordings

EEG signal recorded with the Biosemi ActiveTwo system (Biosemi, Amsterdam, Netherlands) 72 channels at 2048 Hz sampling rate. The Ag/AgCl active EEG electrodes were placed based on the 10–10 standard layout, with two electrodes positioned on the left and right mastoids for references, along with two electrodes for horizontal EOG. ECG electrodes were placed on the collarbone and lower left rib. EDA electrodes were placed on the right palm and wrist. The computer that acquired physiological data via the BioSemi USB Receiver also received X-Plane simulator data, streamed over a dedicated LAN connection at 20 Hz. Each packet of X-Plane and BioSemi data was time-stamped on arrival with the acquisition PC’s system time. This enabled alignment and segmentation of BioSemi data related to X-Plane baseline and trial segments.

The participant’s gaze behaviour was recorded using a Fovio FX3 eye tracker by $Eyeworks^{TM}$^23^, using 60 Hz binocular eye tracking. We positioned the eye tracker under the Primary Flight Display (PFD) and a virtual environment was created using Eyeworks (see Supplementary Figs. S1–S3). This position gave the most reliable results during preliminary testing and reduced gaps in eye-tracking. We calibrated the eye tracker before the start of each experimental session using EyeWorks software using five points on the screen (the middle and the four corners), and then monitored eye tracking accuracy during each maneuver trial. The eye tracker recorded the pilots’ gaze on-screen during the experiment. We set the fixation threshold at 105 ms and $0.5^{\circ }$ of visual angle. Dealing with gaze recordings proves challenging, as the raw data produced by the eye tracker is subject to noise and artifacts. The CTA-toolbox solution implemented in FaubertLab^23^ was used to process the raw data from the eye tracker, interpolate missing data and denoise the recorded traces. The final output of the toolbox was a cleaned-up recording that was used for further analysis. We also recorded participants’ facial expressions and the environment with three cameras for future reference, analysis, and quality control.

### Data analysis

#### Statistical analysis

We performed statistical analysis in JASP^24^. In this study, we assumed each recording variable (e.g. subjective data, EEG) as multiple measures of different task difficulties. If not specified otherwise, all statistics were performed by repeated measures ANOVA and corrected with Holm multiple comparison correction. If sphericity was violated, we used Greenhouse-Geisser for a correction. We used the non-parametric Friedman test to examine the main effects of maneuver difficulty on all the variables because some of them (e.g., D3 in mental demand, D2 in EEG frontal theta) did not meet the normality assumption and we also wanted to compare and unify the results across the variables. If the Friedman test was significant, we performed post hoc tests with Holm correction using the Conover test.

#### Subjective data

After each maneuver, we asked participants to rate their workload using the six sub-scales of the NASA Task Load Index (TLX)^7^. The demand, effort, and frustration sub-scales (1 = very low; 10 = very high) were grouped separately from the performance sub-scale (1 = perfect; 10 = failure), as follows:

- Answer the following questions on a scale from 1 (very low) to 10 (very high):
  - Mental Demand: “How mentally demanding was the task?”
  - Physical Demand: “How physically demanding was the task?”
  - Temporal Demand: “How hurried or rushed was the pace of the task?”
  - Effort: “How hard did you have to work to accomplish your level of performance?”
  - Frustration: “How insecure, discouraged, irritated, stressed, and annoyed were you?”
  - Performance: “On a scale from 1 (perfect) to 10 (failure), how successful were you in accomplishing what you were asked to do?”

#### ECG analysis

In our analysis, an ECG signal was derived as the potential difference between electrodes placed on the right collarbone and the lower left rib. We filtered the signal between 1 and 30 Hz using a Hamming windowed FIR filter in EEGLAB^25^. Then we employed Pan-Tompkins’s algorithm^26^ for pre-processing and R-wave peak detection using a Matlab implementation^27^. Then we calculated R-R intervals (RRI) for each trial and converted R-R intervals to Heart Rate (HR) based on the hyperbolic relationship $HR \times RRI = 60{,}000$.

#### EEG analysis

We analyzed EEG data in EEGLAB^25^ with Matlab (2021b). We processed each block of each participant separately (i.e. 24 participants by two blocks of recording). We down-sampled the signals to 250 Hz, re-referenced all channels to the average of the two mastoid channels, and then band-passed the signals between 0.5 and 55 Hz. We applied Cleanline^28^ to detect and remove the line noises and other single-frequency artifacts caused by recording equipment. Bad channel removal and bad window reconstruction was performed by Artifact Subspace Reconstruction (ASR) algorithm^28^. The Independent component analysis (ICA) was performed after re-referencing to the average. Components were labelled by ICLabel^29^ and those that were related to muscle, eye, and power line artifacts with a confidence interval higher than 85 percent were flagged for removal in the group analysis.

In the group analysis, we interpolated across removed channels and flagged components. We calculated the power spectrum of signals using the Fast Fourier transform and compared the Theta and Alpha bands between task difficulty levels. For the analysis of EEG frontal theta, we selected the mean of F4 and F2 channels as these channels were the most commonly used channels in the current literature^18,20,21,21,22^.

#### Pupil size

We analyzed eye work data in Matlab (2021b). We excluded one session of one participant because the data was corrupted. Inspired by^30^, we removed invalid segments with zero values or low data quality. We discarded signals where the gaze was not directed at the PFD. When the pilot’s head has rotated more than $60^{\circ }$, and the pupil signal was lost, we discarded this gaze direction and all other information during this time segment. When the head remained steady, but the pupil size was lost, we consider it to be blinking. Missing data and blink portions were linearly interpolated^31^. Amplitudes outside the normal range for pupil size (2–8 mm) or two standard deviations away from the mean of trials were labelled as outliers and removed from the data. We filtered the data with a Butterworth second-order band-pass frequency from 1 to 10 Hz and a 5th-order median filter to smooth the signal. For further comparison, we computed the mean of left and right eyes for each task difficulty.

#### Maneuver performance

For each maneuver, we simulated the set of possible changes in altitude and heading that satisfy the instructions. This set accounted for the range of possible accelerations and decelerations to the instructed vertical speed and turn rate that also satisfied the instructed altitude and heading endpoints. The Root Mean Square Error (RMSE) reported for each maneuver trial was the minimum RMSE between actual altitude and heading and the set of idealized altitude and heading profiles for that specific maneuver instruction. Then we combined altitude and heading error by normalizing each trial to the maximum of each participant as follows:

1 $$\begin{aligned} \text {Normalized RMSE}(i) = 0.5 * \frac{log_{10} (\text {Altitude RMSE}(i))}{max(log_{10} (\text {Altitude RMSE})} + 0.5 * \frac{log_{10} (\text {Heading RMSE}(i))}{max(log_{10}(\text {Heading RMSE})} \end{aligned}$$

where index *i* represents an individual maneuver trial.

### Supplementary Information

Supplementary Information.

## Data Availability

The datasets analyzed during the current study are available from the corresponding author upon reasonable request.
