# Supplementary material for: Beyond performance: the role of task demand, effort, and individual differences in ab initio pilots
Source: Sci Rep. 2023 Aug 28;13:14035. doi: 10.1038/s41598-023-41427-4 (PMC10462656; doi:10.1038/s41598-023-41427-4)
Supplement: Supplementary file 1 — Supplementary Information. [file 41598_2023_41427_MOESM1_ESM.pdf]

Appendices

Maneuver Instructions

- D1 manuever instructions:
  - D1.A: Maintain altitude at 10,000 feet. At the same time: Turn LEFT at 30 deg AOB to a heading of 240 degrees and roll out on a steady heading.
  - D1.B: Maintain altitude at 10,000 feet. At the same time: Turn RIGHT at 30 deg AOB to a heading of 120 degrees and roll out on a steady heading.
  - D1.C: Maintain heading at 0 degrees. At the same time: CLIMB to an altitude of 11,000 feet at 1000 fpm and level off.
  - D1.D: Maintain heading at 0 degrees. At the same time: DESCEND to an altitude of 9,000 feet at 1000 fpm and level off.
- D2 manuever instructions:
  - D2.A: Maintain altitude at 10,000 feet. At the same time: Turn LEFT at 30 deg AOB to a heading of 300 degrees; Then turn RIGHT at 30 deg AOB back to a heading of 0 degrees and roll out on a steady heading.
  - D2.B: Maintain altitude at 10,000 feet. At the same time: Turn RIGHT at 30 deg AOB to a heading of 60 degrees; Then turn LEFT at 30 deg AOB back to a heading of 0 degrees and roll out on a steady heading.
  - D2.C: Maintain heading at 0 degrees. At the same time: CLIMB to an altitude of 10500 feet at 1000 fpm; Then DESCEND back to an altitude of 10,000 feet at 1000 fpm and level off.
  - D2.D: Maintain heading at 0 degrees. At the same time: DESCEND to an altitude of 9500 feet at 1000 fpm; Then CLIMB back to an altitude of 10,000 feet at 1000 fpm and level off.
- D3 manuever instructions:
  - D3.A: DESCEND to an altitude of 9,000 feet at 1000 fpm and level off. At the same time: Turn RIGHT at 30 deg AOB to a heading of 60 degrees; Then turn LEFT at 30 deg AOB back to a heading of 0 degrees and roll out on a steady heading.
  - D3.B: CLIMB to an altitude of 11,000 feet at 1000 fpm and level off. At the same time: Turn RIGHT at 30 deg AOB to a heading of 60 degrees; Then turn LEFT at 30 deg AOB back to a heading of 0 degrees and roll out on a steady heading.
  - D3.C: DESCEND to an altitude of 9,000 feet at 1000 fpm and level off. At the same time: Turn LEFT at 30 deg AOB to a heading of 300 degrees; Then turn RIGHT at 30 deg AOB back to a heading of 0 degrees and roll out on a steady heading.
  - D3.D: CLIMB to an altitude of 11,000 feet at 1000 fpm and level off. At the same time: Turn LEFT at 30 deg AOB to a heading of 300 degrees; Then turn RIGHT at 30 deg AOB back to a heading of 0 degrees and roll out on a steady heading.
  - D3.E: Turn LEFT at 30 deg AOB to a heading of 240 degrees and roll out on a steady heading. At the same time: CLIMB to an altitude of 10500 feet at 1000 fpm; Then DESCEND back to an altitude of 10,000 feet at 1000 fpm and level off.
  - D3.F: Turn RIGHT at 30 deg AOB to a heading of 120 degrees and roll out on a steady heading. At the same time: CLIMB to an altitude of 10500 feet at 1000 fpm; Then DESCEND back to an altitude of 10,000 at 1000 fpm and level off.
  - D3.G: Turn LEFT at 30 deg AOB to a heading of 240 degrees and roll out on a steady heading. At the same time: DESCEND to an altitude of 9500 feet at 1000 fpm; Then CLIMB back to an altitude of 10,000 feet at 1000 fpm and level off.
  - D3.H: Turn RIGHT at 30 deg AOB to a heading of 120 degrees and roll out on a steady heading. At the same time: DESCEND to an altitude of 9500 feet at 1000 fpm; Then CLIMB back to an altitude of 10,000 feet at 1000 fpm and level off.

Correlation Analysis

The Spearman's rho correlation between different metrics was calculated in JASP and colour-coded from blue to red. Significant correlations are highlighted with * p < .05, ** p < .01, p < .001. Because the independency assumption might be violated as this is a repeated measure experiment[1], we used Repeated measure Correlation (RMCorr) which considers individual means[1] in Supplementary Figure S1.

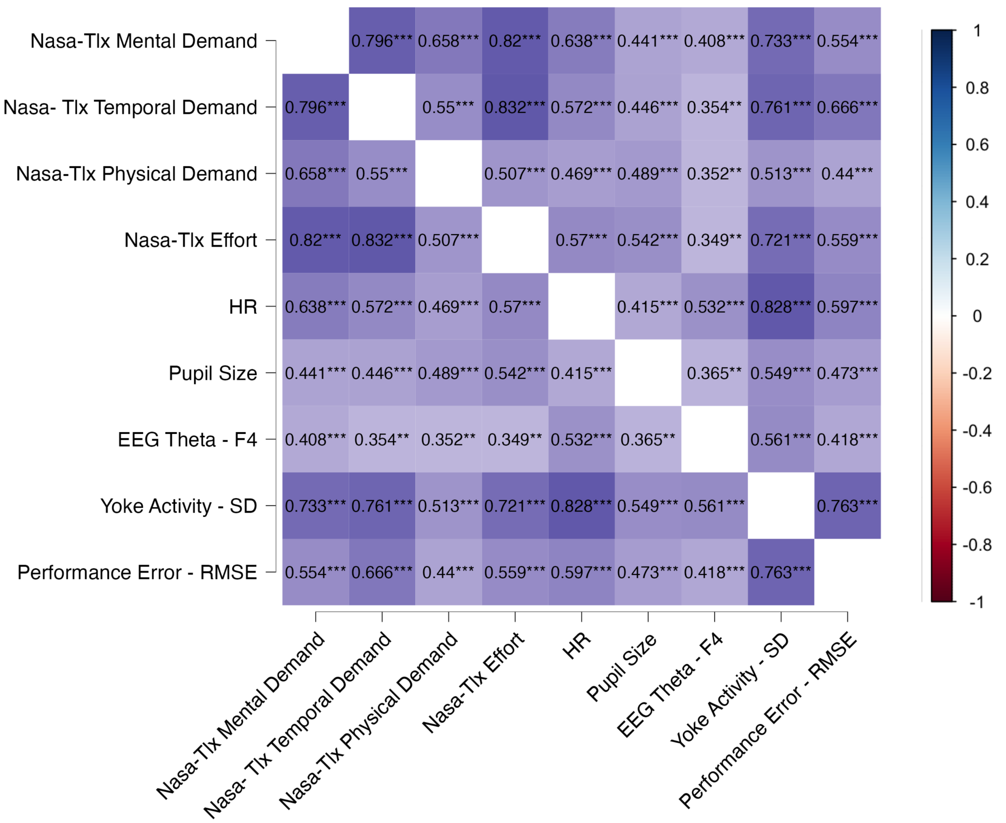

Supplementary figure S1. Heatmap of repeated measures correlation between all measures with significant main effects of task difficulty. The Spearman's rank correlation coefficient between different metrics is calculated and colour-coded from blue to red. Significant correlations are highlighted with * p < .05, ** p < .01, p < .001.

EEG Theta and Alpha

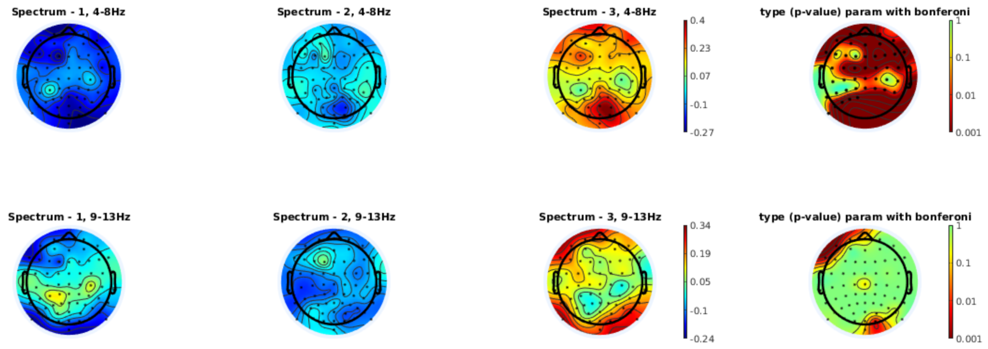

Supplementary figure S2. EEG activity in the theta and alpha frequency bands across task difficulty levels. The brain maps show EEG theta activities for each task level (1, 2 and 3). The scale indicators for colour maps are on the right side of the third and fourth maps.

We provide a global view of brain activity in the theta and alpha frequency bands between task difficulty levels. The brain maps in Supplementary Figure S2 show EEG theta activities for each task level (1, 2 and 3). The scale indicators for colour maps are on the right side of the third and fourth maps. As can be seen, the theta activity increases with the task difficulty level, especially in the frontal regions. In the main paper, we only used the theta band of two EEG frontal channels (F2 and F4) as these channels were frequently mentioned in the literature as correlated with workload. These channels showed significant differences between task levels and correlated with the performance measures.

Eye tracker setup

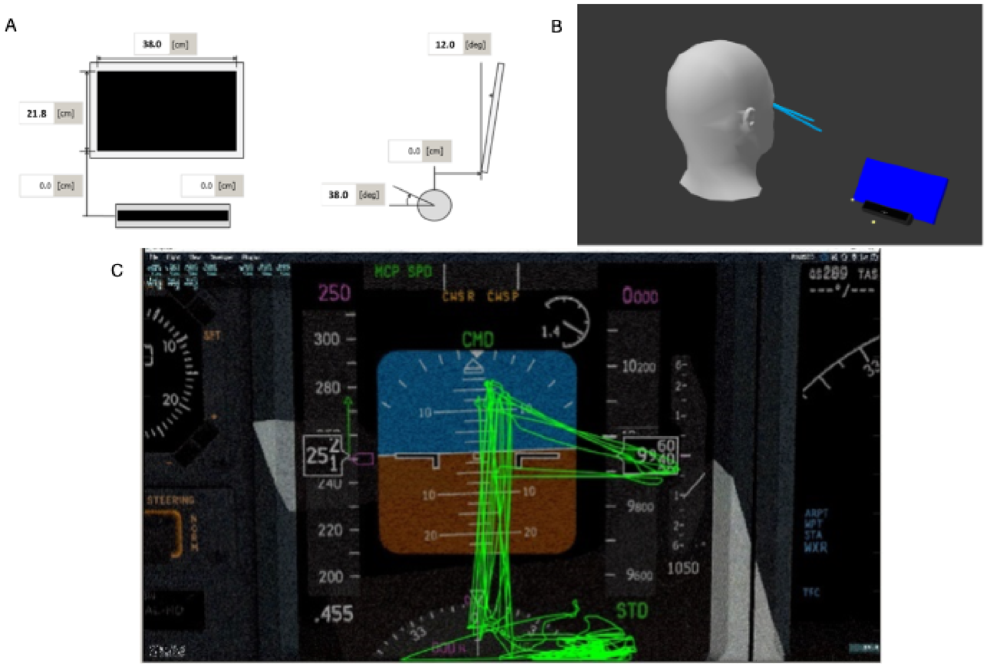

Supplementary figure S3. Eye tracker setup. A) The display and eye tracker diagram in front and side view. B) Point of view of participants. C) A sample of eye gazes at the display.

References

1. Bakdash, J. Z. & Marusich, L. R. Repeated measures correlation. *Front. psychology* **8**, 456 (2017).
